# Supplementary material for: Astragalus Saponins, Astragaloside VII and Newly Synthesized Derivatives, Induce Dendritic Cell Maturation and T Cell Activation
Source: Vaccines (Basel). 2023 Feb 21;11(3):495. doi: 10.3390/vaccines11030495 (PMC10059926; doi:10.3390/vaccines11030495)
Supplement: Supplementary file 1 [file vaccines-11-00495-s001.zip › vaccines-2177245-supplementary.pdf]

Supporting Information

Astragalus Saponins, Astragaloside VII and Newly Synthesized Derivatives, Induce Dendritic Cell Maturation and T Cell Activation

Nilgun Yakubogullari[1], Ali Cagir[2], Erdal Bedir[1], Duygu Sag[3,4,5]

[1]Izmir Institute of Technology, Department of Bioengineering, Izmir, Turkey

[2]Izmir Institute of Technology, Department of Chemistry, Izmir, Turkey

[3]Izmir Biomedicine and Genome Center, Izmir, Turkey

[4]Izmir International Biomedicine and Genome Institute, Dokuz Eylul University, Izmir, Turkey

[5]Department of Medical Biology, Faculty of Medicine, Dokuz Eylul University, Izmir, Turkey

*Address correspondence and reprint requests to Duygu Sag (duygu.sag@ibg.edu.tr)

Table S1. Semi-synthesis of AST VII Analogs (DC-AST VII and DAC-AST VII) and structure elucidation by NMR and MS

Semi-synthesis protocol	^{1}H NMR (400 MHz)	^{13}C NMR (100 MHz)
**DC-AST VII :**<br>AST VII (1000 mg, 1.06 mmol, 1 equiv.), NaBr (109 mg, 1.06 mmol, 1 equiv.) and TEMPO (40 mg, 0.212 mmol, 0.2 equiv.) were dissolved in distilled water (pH 11 adjusted with 1 N NaOH). NaOCl (6.3 mL, 4.66 mmol, 4.4 equiv) was slowly added to the reaction mixture and stirred at 0°C. After 4 h, the reaction was quenched by addition of distilled water and neutralized by 1 M HCl. Reaction mixture was extracted (x3) with *n*-butanol and water phase was evaporated at 50°C in rotary evaporator. Further purification was done by VLC (vacuum liquid chromatography) loaded with reversed-phase silica gel (RP-18, 25 g) using MeOH/H_2O gradient (15:85, 20:80, 35:65, 40:60; 50:50, 55:45, 100:0).<br>**TOF-MS m/z:** 995.38897 ([M+Na-2H]$^{-}$), $C_{47}H_{72}O_{21}Na$= 995.44638	**^{1}H-NMR (D_2O)** δ 4.72 (1H, dd, *J= 6, 4.5 Hz*, H-16), 4.70 (1 H, d, *J= 8.1 Hz*, H-1'''), 4.56 (1 H, d, *J= 7.9 Hz*, H-1''), 4.50 (1 H, d, *J= 7.8 Hz*, H-1'), 3.97 (1 H, dd, *J= 15.5, 6.4 Hz*, H-24), 3.96 (1 H, d, *J= 5.8 Hz*, H-5'), 3.73 (2 H, s, H-5'', H-5'''), 3.69 (1 H, d, *J= 5.3 Hz*, H-6), 3.68 (1 H, d, *J= 9.1 Hz*, H-4'), 3.56 (1 H, d, *J= 4.1 Hz*, H-4''), 3.54 (1 H, d, *J= 2.7 Hz*, H-3'), 3.52 (1 H, d, *J= 2.9 Hz*, H-3''), 3.50 (1 H, s, H-3'''), 3.39 (2 H, d, *J= 4.4 Hz*, H-2', H-3), 3.33 (1 H, s, H-5'), 3.31 (1 H, d, *J= 6.2 Hz*, H-2''), 3.30 (1 H, d, *J= 8.5 Hz*, H-2'''), 3.56 (1 H, d, *J= 4.1 Hz*, H-4'''), 2.46 (2 H, d, *J= 8 Hz*, H-17, H-22), 2.16 (1 H, s, H-23), 2.12 (2 H, d, *J= 9.2 Hz*, H-23, H-11), 2.08 (1 H, m, H-15), 1.97 (1 H, d, *J= 7.7 Hz*, H-2), 1.95 (1 H, d, *J= 7.7 Hz*, H-7), 1.84 (1 H, d, *J= 12.2 Hz*, H-8), 1.75 (1 H, d, *J= 12.5 Hz*, H-22), 1.74 (1 H, d, *J= 12.5 Hz*, H-12), 1.68 (1 H, s, H-5), 1.67 (2 H, s, H-1, H-2), 1.64 (1 H, s, H-12), 1.45 (1 H, d, *J= 10.1 Hz*, H-7), 1.44 (1 H, d, *J= 10.1 Hz*, H-15), 1.39 (3 H, s, H-26), 1.32 (3 H, s, H-28), 1.30 (3 H, s, H-27), 1.29 (6 H, s, H-21, H-18), 1.22 (1 H, s, H-1), 1.16 (1 H, s, H-11), 1.06 (3 H, s, H-29), 1.01 (3 H, s, H-30), 0.7 (1 H, s, H-19), 0.4 (1 H, s, H-19).	**^{13}C-NMR (D_2O)** δ 176.1 (s, C-6''), 175.7 (s, C-6'''), 105.5 (d, C-1'), 102.5 (d, C-1''), 96.8 (d, C-1'''), 89.3 (d, C-3), 87.9 (s, C-20), 81.6 (d, C-24), 80.3 (d, C-6), 79.7 (s, C-25), 76.6 (d, C-5''), 76.3 (d, C-5'''), 76.1 (d, C-3'), 75.9 (d, C-3'', C-3'''), 73.8 (d, C-16), 73.7 (d, C-2'''), 73.6 (d, C-2'), 73.1 (d, C-2''), 71.8 (d, C-4'', C-4'''), 69.4 (d, C-4'), 65.1 (t, C-5'), 57.0 (d, C-17), 51.6 (d, C-5), 46.3 (d, C-8), 45.6 (s, C-13), 44.8 (s, C-14), 44.2 (t, C-15), 41.5 (s, C-4), 34.6 (t, C-7), 34.3 (t, C-22), 32.9 (t, C-12), 31.9 (t, C-1),), 30.7 (t, C-19), 29.2 (t, C-2), 29.1 (s, C-10), 27.4 (q, C-28), 27.3 (q, C-21), 25.7 (t, C-11), 25.6 (t, C-23), 21.2 (q, C-18), 20.4 (s, C-9), 24.7 (q, C-26), 21.5 (q, C-27), 19.3 (q, C-30), 15.7 (q, C-29).
**DAC-AST VII:**<br>Dicarboxylic AST-VII (50 mg, 0.0513 mmol, 1 equivalent) was dissolved in pyridine. DIPEA (27 mg, 0.2052 mmol, 4 equiv.), HOBt (16 mg, 0.1026 mmol, 2 equiv.), EDC (30 mg, 0.1539 mmol, 3 equiv.) were added and the reaction mixture was stirred for 1 h at room temperature. 1 h later, free dodecylamine (23 mg, 0.1231 mmol, 2.4 equiv.) was added dropwise and reaction mixture was heated to 60°C. After 6 h, the reaction was quenched by addition of distilled water and extracted (x3) with ethyl acetate. Ethyl acetate fraction was evaporated at 50°C in rotary evaporator. DAC-AST VII was purified by silica gel open-column chromatography (30 g) eluting with $CHCl_3$: MeOH: H_2O (80:20:2).<br>**TOF-MS m/z:** 1331.89196 ([M+Na]$^{+}$), $C_{71}H_{124}O_{19}Na$ =1331.86960	**^{1}H-NMR (400 MHz, d5)** δ 8.13 (1H, t, *J = 6.2 Hz*, H-1^{v}), 8.09 (1H, t, *J = 6.1 Hz*, H-1^{iv}), 5.12 (1H, d, *J = 7.8 Hz*, H-1'''), 4.99 (1H, s, H-16), 4.95 (1H, d, *J = 7.8 Hz*, H-1''), 4.86 (1H, d, *J =7.4 Hz*, H-1'), 4.36 (1H, dd, *J = 11.0, 4.7 Hz*, H-5'), 4.35 (1H, s, H-5''), 4.35 (1H, d, *J = 5.3 Hz*, H-5'''), 4.26 (2H, dd, *J = 7.8, 4.7 Hz*, H-3'', H-4''), 4.26 (1H, m, H-2'''), 4.22 (1H, m, H-4'), 4.21 (2H, d, *J = 4.9 Hz*, H-3''', H-4'''), 4.15 (1H, t, H-3'), 4.04 (2H, m, H-2', H-2''), 3.95 (1H, dd, *J = 8.5, 6.1 Hz*, H-24), 3.83 (1H, m, H-6), 3.72 (1 H, d, *J = 10.7 Hz*, H-5'), 3.64 (2H, m, H-2^{v}), 3.53 (1H, dd, *J = 11.8, 4.4 Hz*, H-3), 3.46 (2H, m, H-2^{iv}), 2.76 (1H, t, *J = 9.9 Hz*, H-22),2.53 (1H, m, H-17), 2.39 (1H, d, *J = 12.4 Hz*, H-11), 2.34 (1H, s, H-23), 2.26 (1H, dd, *J = 12.3, 7.9 Hz*, H-15),2.15 (1H, dd, *J = 8.5, 3.5 Hz*, H-7), 2.01 (1H, s, H-8), 1.99 (3H, s, H-28), 1.95 (1H, m, H-23), 1.95 (1H, s, H-11), 1.88 (1H, d, *J = 8.8, 4.4 Hz*, H-5), 1.84 (1H, d, *J = 10.1*, H-7), 1.80 (1H, m, H-1), 1.78 (1H, d, *J = 6.4 Hz*, H-15), 1.74 (1H, d, *J = 7.3 Hz*, H-12), 1.73 (1H, m, H-2), 1.65 (1 H, s, H-22), 1.64 (3H, s, H-26), 1.62-1.82 (36H, m, H-3^{iv}-11^{iv}, H-3^{v}-11^{v}), 1.56 (2H, d, *J = 12.1 Hz*, H-1, H-12), 1.44 (3H, s, H-27), 1.42 (3H, s, H-18), 1.37 (3H, s, H-29), 1.32 (3H, s, H-21), 1.29 (4H, m, H-12^{iv}, H-12^{v}), 1.26 (1H, m, H-2), 1.12 (3H, s, H-30), 0.88 (6H, s, H-13^{iv}, H-13^{v}), 0.61 (1H, d, *J = 3.9 Hz*, H-19), 0.25 (1H, d, *J = 4.1 Hz*, H-19).	**^{13}C-NMR (100 MHz, d5)** δ 171.5 (s, C-6''), 171.4 (s, C-6'''), 108.2 (d, C-1'), 105.2 (d, C-1''), 99.1 (d, C-1'''), 88.9 (d, C-3), 87.7 (s, C-20), 82.5 (d, C-24), 79.7 (s, C-25), 79.5 (d, C-6), 79 (d, C-3'), 78.9 (d, C-3'', C-2'''), 78.3 (d, C-3'''), 76.6 (d, C-5''), 76.4 (d, C-5'''), 75.4 (d, C-2''), 74.9 (d, C-2'), 74.4 (d, C-4'''), 74.2 (d, C-4''), 74.1 (d, C-16), 71.8 (d, C-4'), 67.6 (t, C-5'), 58.6 (d, C-17), 52.9 (d, C-5), 46.8 (s, C-13), 46.7 (t, C-15), 45.9 (d, C-8), 45.9 (s, C-14), 43.2 (s, C-4), 39.8 (t, C-2^{v}), 35.7 (t, C-22), 34.7 (t, C-7), 33.9 (t, C-12), 32.7 (t, C-1,C-12^{iv},C-12^{v}), 30.2 (t, C-2), 30-31 (t, C-3^{v}- C-11^{v}), 29.5 (d, C-10), 28.9 (t, C-19), 28.2 (q, C-28), 27.8 (q, C-21), 27.8-31 (t, C-3^{iv}- C11^{iv}), 26.7 (t, C-11), 26.5 (t, C-23), 25.4 (q, C-26), 23.5 (q, C-27), 21.6 (q, C-18), 21.5 (s, C-9), 20.6 (q, C-30), 17.1 (q, C-29), 14.6 (q, C-13^{iv}, C-13^{v}).

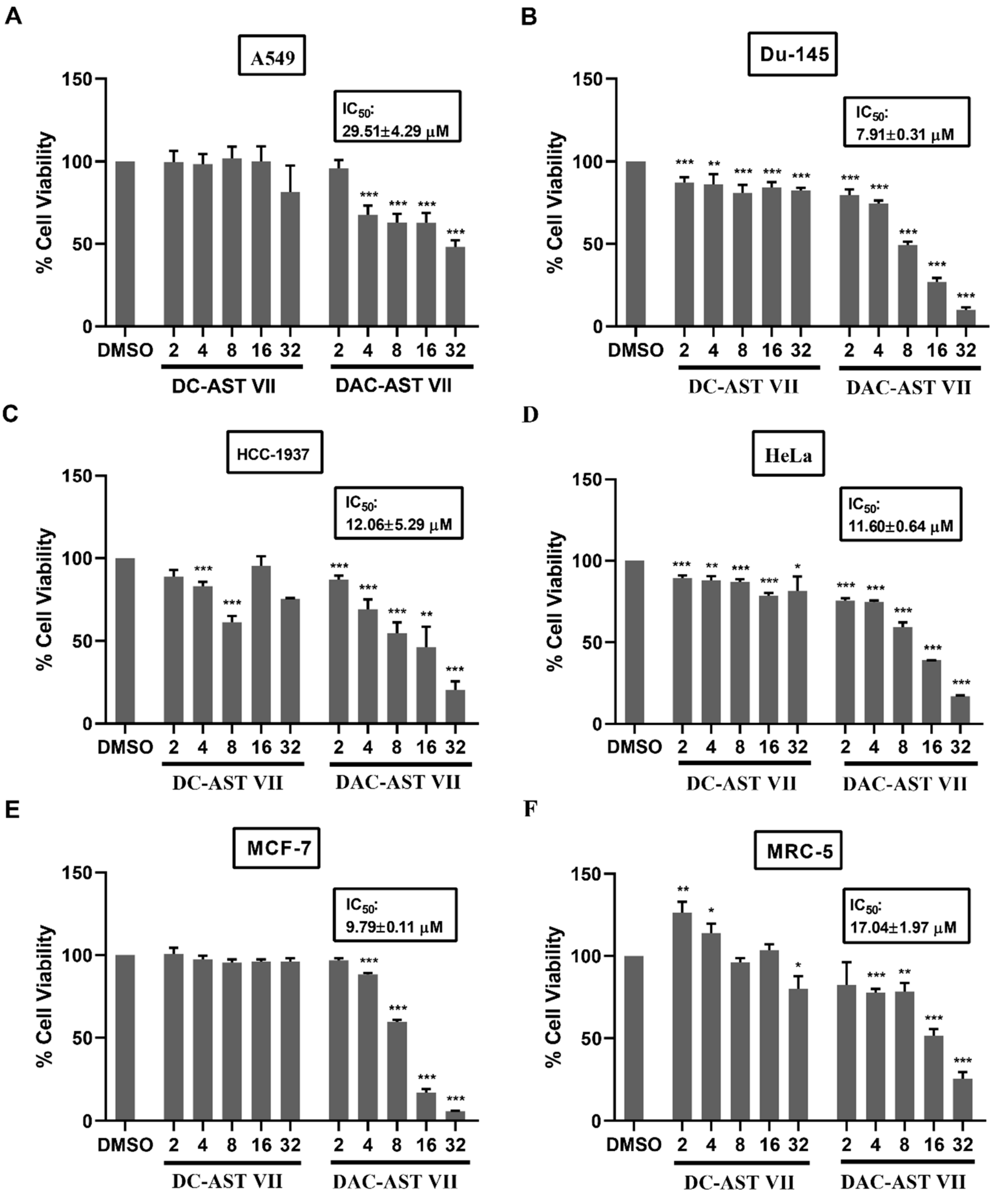

Figure S1. Determination of cell viability and IC_{50} values in cancer and healthy cell lines treated with DC-AST VII and DAC-AST VII. The cell viability (%) and related IC_{50} values in different cancer (A) A549, (B) Du145, (C) HCC-1937, (D) HeLa, (E) MCF-7 and healthy (F) MRC-5 cell lines treated with DC-AST VII and DAC-AST VII were represented. A549, Du145, HCC-1937, HeLa, MCF-7 and MRC-5 cell lines were treated with DC-AST VII and DAC-AST VII at the concentrations of 2 to 32 μM for 48 h. The cell viability was analyzed by MTT assay and calculated compared to DMSO. DMSO alone was used as vehicle control, with the results representing average values from individual experiment, each performed in triplicate. Statistical analyses were performed between treated groups

and DMSO control using One-way ANOVA and Dunnett's multiple comparisons test. *$p < 0.05$, **$p < 0.01$, ***$p < 0.001$.

Table S2. Hemolytic activity of DAC-AST VII and DC-AST VII. Statistically significant differences of treated groups versus saline control are indicated ***$p < 0.001$ by one-way ANOVA and multiple comparion test.

Groups	Hemolysis (%)
Saline	0
Distilled Water	100***
DAC-AST VII (μM)	
250	1,377 ± 0,031
50	2,411 ± 1,922
10	1,436 ± 0,658
2	0,762 ± 0,053
0.4	1,199 ± 0,163
DC-AST VII (μM)	
250	1,246 ± 0,553
50	0,763 ± 0,012
10	1,616 ± 0,897
2	0.755 ± 0,204
0.4	1,413 ± 0,396

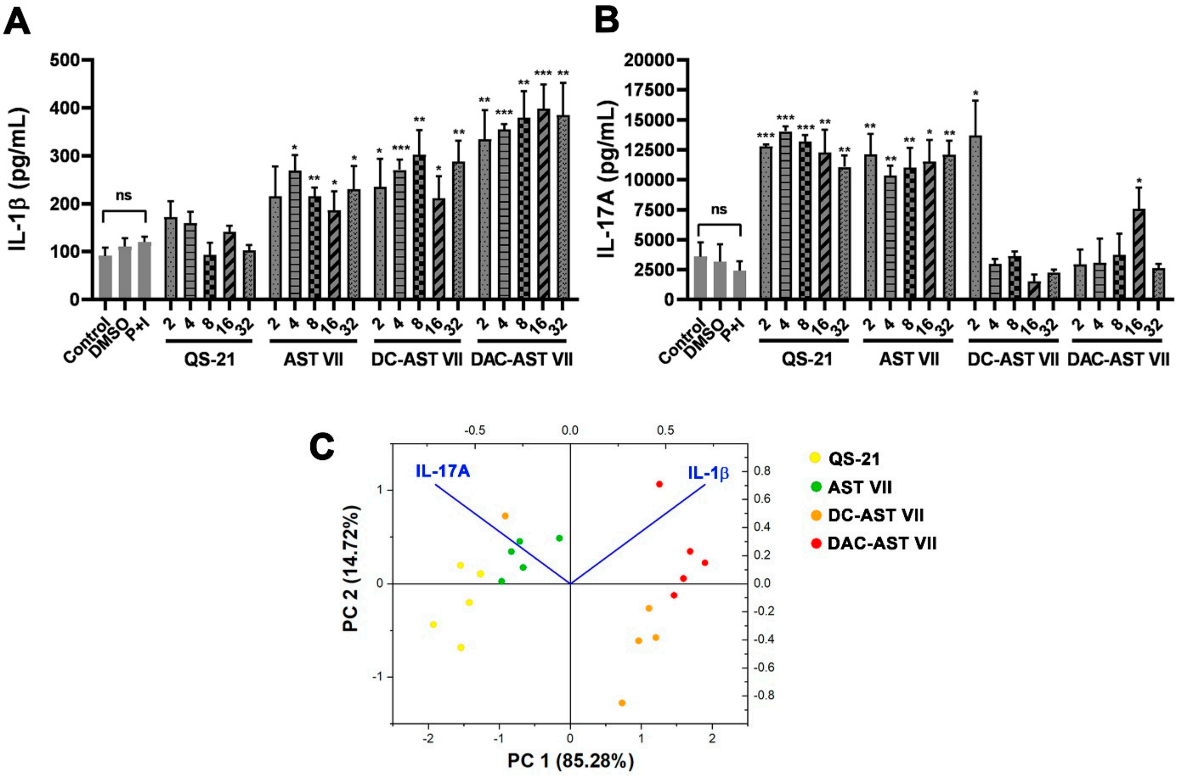

Figure S2. AST VII and its derivatives (DC-AST VII and DAC-AST VII) alter the production of pro-inflammatory cytokines in 1/10 diluted hWB cells. 1/10 diluted hWB was co-treated with PMA (50 ng/mL)/ionomycin (400 ng/mL) and the following compounds QS-21, AST VII, DC-AST VII, DAC-AST VII at the concentration of 2-32 μg/mL for 48 h. The supernatants were collected for the detection of **(A)** IL-1β and **(B)** IL-17A by ELISA. **(C)** Principles component analysis projected onto the plane of the first two principal components (PCs) and colored by the different compounds. Data shown are mean ± SD of triplicate determinations and representative of two independent experiments with similar results. Statistical analyses were performed between control and P+I (PMA+Ionomycin) by Student-t test and, P+I and treated groups by One-way ANOVA and Tuk-ey's multiple comparison tests. *$p < 0.05$, **$p < 0.01$, ***$p < 0.001$, not statistically significant (ns).

Table S3. Principle component (PC) loadings for each cytokine. The loadings demonstrated the correlation between each cytokine and PC.

1/10 Diluted hWB	PC1	PC2
IL-1β	0.707	0.707
IL-17A	-0.707	0.707

1/20 Diluted hWB	PC1	PC2
IL-1β	0.506	-0.552
TNF-α	0.568	0.183
IL-2	0.121	0.804
IFN-γ	0.637	0.124

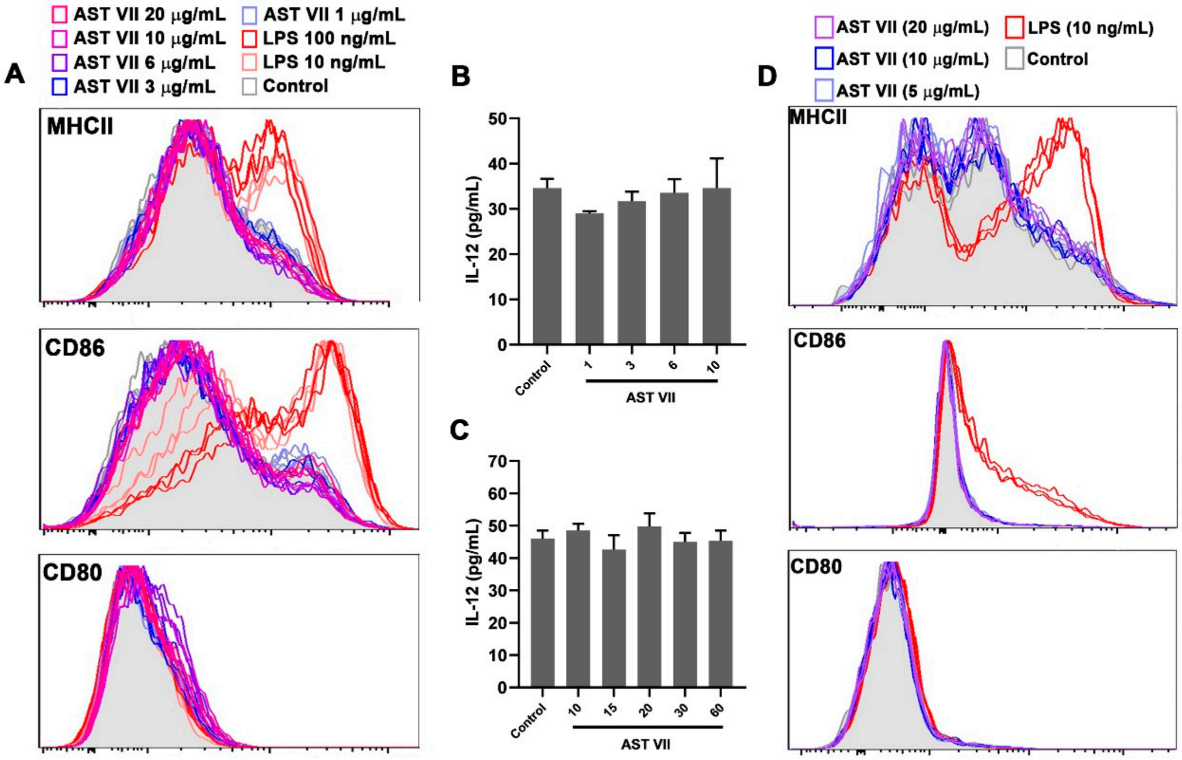

Figure S3. AST VII alone did not induce the maturation and activation of BMDCs and splenic dendritic cells. BMDCs or splenocytes were treated with AST VII at the concentrations of 1 to 20 μg/mL for 24 h. The surface marker expression (**A** and **D**) by BMDCs and $CD11c^{+}MHCII^{+}$ dendritic cells in splenocytes were analyzed by flow cytometry. (**B** and **C**) IL-12 titers in the cell culture supernatant of BMDCs were measured by ELISA. Representative data from one of the two independent experiments, each performed in triplicate, are shown.

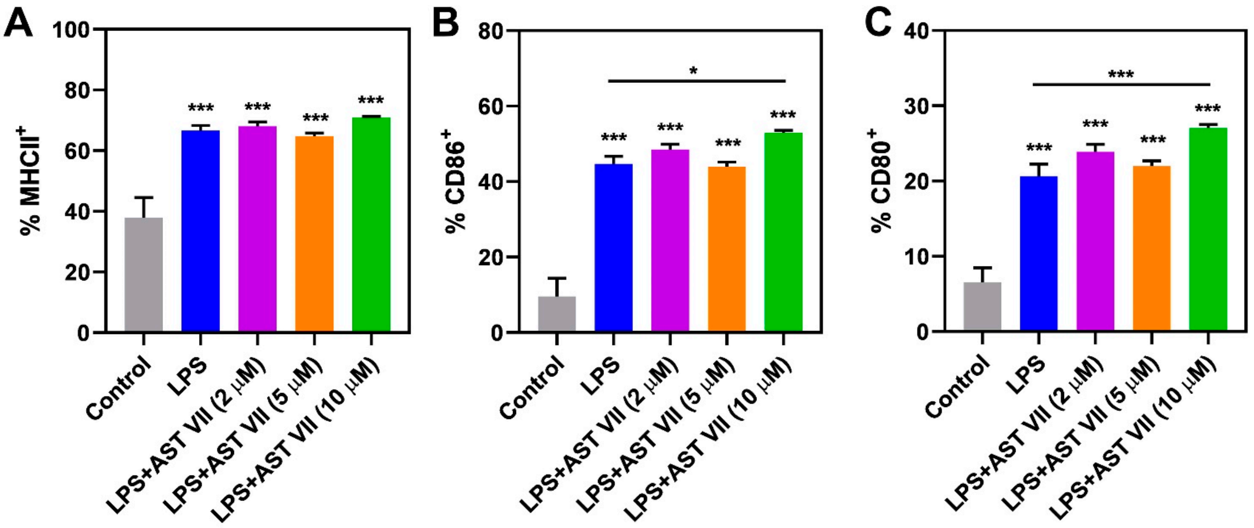

Figure S4. AST VII induces the maturation and activation of BMDCs in the presence of LPS. Bar graphs show the frequencies of **(A)** MHCII^{+}, **(B)** CD86^{+}, **(C)** CD80^{+} BMDCs. Data are representative of two independent experiments with similar results. Statistical analyses were performed between treated groups and control (untreated cells) using One-way ANOVA and Tukey's multiple comparisons test. *$p < 0.05$, ***$p < 0.001$.

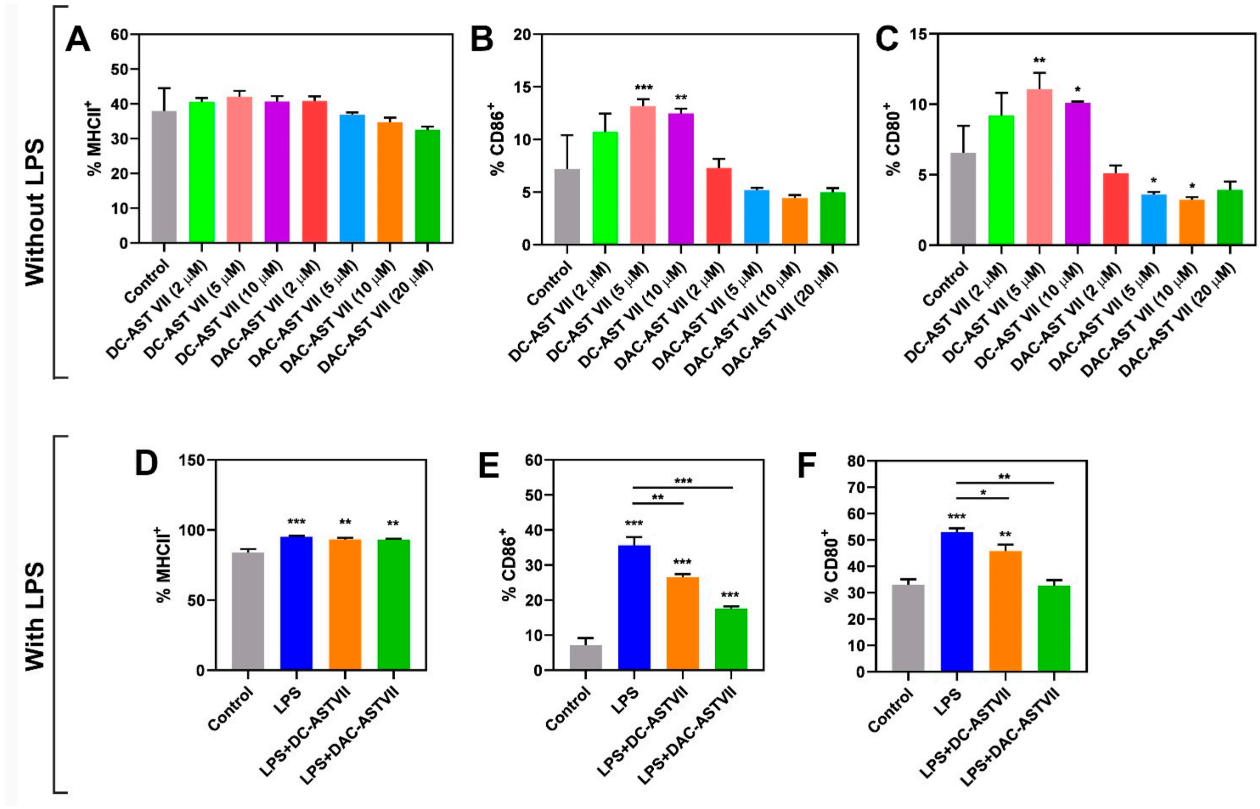

Figure S5. The effect of AST VII derivatives (DC-AST VII and DAC-AST VII) on the maturation and activation of BMDCs in the absence/ presence of LPS. Bar graphs show the frequencies of **(A)** MHCII$^+$, **(B)** CD86$^+$, **(C)** CD80$^+$ BMDCs treated with DC-AST VII and DAC-AST VII in the absence of LPS and the frequencies of **(D)** MHCII$^+$, **(E)** CD86$^+$, **(F)** CD80$^+$ BMDCs treated with DC-AST VII and DAC-AST in the presence of LPS. Data are representative of two independent experiments with similar results. Statistical analyses were performed between treated groups and control (untreated cells) using One-way ANOVA and Tukey's multiple comparisons test. *$p < 0.05$, **$p < 0.01$, ***$p < 0.001$.

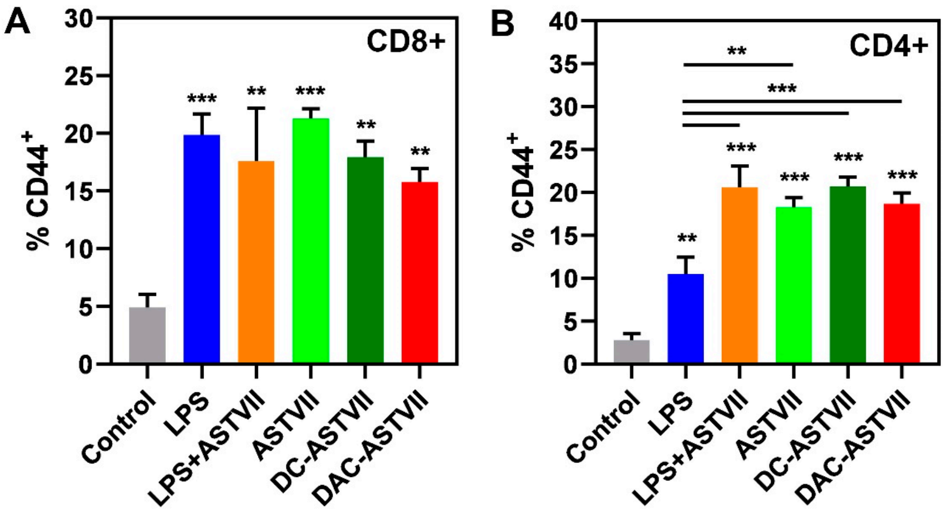

Figure S6. AST VII and its derivatives (DC-AST VII and DAC-AST VII) activated T cells in MLR. Bar graphs show the frequencies of $CD44^+$ **(A)** $CD8^+$ T cells and **(B)** $CD4^+$ T cells. Data are representative of two independent experiments with similar results. Statistical analyses were performed between treated groups and control (untreated cells) using One-way ANOVA and Tukey's multiple comparisons test. $^{**}p < 0.01$, $^{***}p < 0.001$.
